# Irving-Williams Order in the Framework of Connectivity Index ^3^*χ^v^* Enables Simultaneous Prediction of Stability Constants of Bivalent Transition Metal Complexes

**DOI:** 10.3390/molecules16021103

**Published:** 2011-01-26

**Authors:** Ante Miličević, Gina Branica, Nenad Raos

**Affiliations:** Institute for Medical Research and Occupational Health, Ksaverska c. 2, P. O. Box 291, HR-10001, Zagreb, Croatia

**Keywords:** amino acid chelates, topological indices, regression models

## Abstract

Logarithms of stability constants, log *K*_1_ and log *β*_2_, of the first transition series metal *mono*- and *bis*-complexes with any of four aliphatic amino acids (glycine, alanine, valine and leucine) decrease monotonously with third order valence connectivity index, ^3^*χ^v^*, from Cu^2+^ to Mn^2+^. While stability of the complexes with the same metal is linearly dependent on ^3^*χ^v^*, stability constants of Mn^2+^, Fe^2+^, Co^2+^, and Ni^2+^complexes with the same ligand show a quadratic dependence on ^3^*χ^v^*. As Cu^2+^ complexes deviate significantly from quadratic functions, models for the simultaneous estimation of the stability constants, yielding *r* = 0.999 (S.E. = 0.05) and *r* = 0.998 (S.E. = 0.11), for log *K*_1_ and log *β*_2_, respectively, were developed only for Mn^2+^, Fe^2+^, Co^2+^, and Ni^2+^ complexes with amino acids.

## 1. Introduction

The Irving-Williams order of stability of bivalent transition metal complexes (Mn^2+^<Fe^2+^<Co^2+ ^<Ni^2+^<<Cu^2+^>Zn^2+^) [1,2] is an empirical rule well known to every chemist, but it was rarely used for the quantitative prediction of stability constants. Cannon developed an interpolation formula [3] to predict stability constants of chromium(II) complexes from the constants of copper(II), manganese(II), and zinc(II), but it was later found to be in no way better than similar formulas based on one variable, namely stability constants of copper(II), or merely protonation constant of the ligand [4]. 

In our systematic attempt to develop regression models based on the third order valence connectivity index (^3^*χ^v^*) for the prediction of stability constants of coordination compounds [5,6], we were concerned mostly with the copper(II) and nickel(II) chelates. We developed models not only for the complexes of the same metal, but also the simultaneous prediction of stability constants of copper(II) and nickel(II). This was done by introducing an indicator variable, *i.e.* assuming that the regression lines for the two metals have the same slope [7,8]. 

Connectivity, as well as other topological indices [9,10], have found wide range of application in all fields of chemisty [11,12,13,14], but they were not used for the prediction of stability constants of coordination compounds before the appearance of our pioneering work in 1999 [15]. Even now, many chemists are reluctant to use such a simple method, as models based on topological indices really are, to solve such a complex problem as prediction of stability of coordination compounds. 

However, regression models based on valence connectivity index of the third order are capable of predicting stability constants with an error usually less than 0.3 log *K* units, and were even successfully used for the evaluation of experimental data obtained by two different electrochemical methods, potentiometry and voltammetry [16]. The regression models were successfully checked on complexes of *α*-amino acids and their *N*-alkylated derivatives [16,17], amines [17,18,19] and smaller peptides from dipeptides to pentapeptides [7,20]. Also, we recently applied our model to cadmium(II) complexes with amino acids [21].

The aim of this paper is to make our regression models based on ^3^*χ^v^* index more general, *i.e.* to develop models that would discriminate not only ligands, but metals as well. As complexes of the metals from Irving-Williams order seems ideal for this purpose, we choose stability constants of their *mono*-

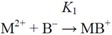
(1)
and *bis*-complexes

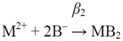
(2)
with aliphatic *α*-amino acids (M stays for metal and B for ligand).

## 2. Methods

### 2.1. Calculation of topological indices

We calculated topological indices using a program system E-DRAGON, developed by Todeschini and coworkers [22,23], which is capable of yielding 119 topological indices in a single run, along with many other molecular descriptors [24,25]. Connectivity matrices were constructed with the aid of the *Online SMILES Translator and Structure File Generator* [26]. All models were developed by using ^3^*χ^v^* index (the valence molecular connectivity index of the 3^rd^ order), which was defined as [27,28,29]:

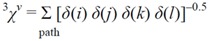
(3)
where *δ*(*i*), *δ*(*j*), *δ*(*k*), and *δ*(*l*) are weights (valence values) of vertices (atoms) *i*, *j*, *k*, and *l* making up the path of length 3 (three consecutive chemical bonds) in a vertex-weighted molecular graph.Valence value, *δ*(*i*), of a vertex *i* is defined by:

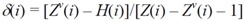
(4)
where *Z^v^*(*i*) is the number of valence electrons belonging to the atom corresponding to vertex *i*, *Z*(*i*) is its atomic number, and *H*(*i*) is the number of hydrogen atoms attached to it. For instance, *δ* values for primary, secondary, tertiary and quaternary carbon atoms are 1, 2, 3, and 4, respectively; for oxygen in the OH group it equals 5, and for NH_2_ group *δ*(N) = 3. It has to be pointed out that ^3^*χ^v^* is only a member of the family of valence connectivity indices *^n^χ^v^*, which differ between each other by the path length, *i.e.* the number of *δ´* sin the summation term, Equation (3).

**Figure 1:**
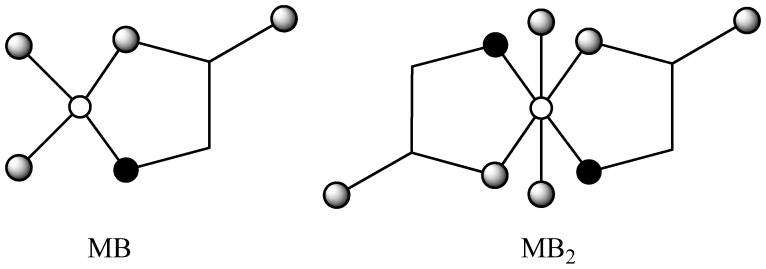
The graph representations for metal(II) *mono*- (MB) and *bis*-complex (MB_2_) with glycine. Heteroatoms are marked with 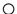 (M), 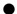 (N), and 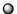 (O).

The ^3^*χ^v^* indices for all *mono*- and *bis*-complexes were calculated from the graph representations of the *aqua* complexes with two water molecules (Figure 1), assuming that metal in *mono*-complexes is tetracoordinated, and in *bis*-complexes hexacoordinated [17,30]. 

### 2.2. Regression calculations

Regression calculations, including the leave-one-out procedure of cross validation, cv, were done using the CROMRsel program [31]. The standard error of cross validation estimate is defined as:

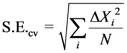
(5)
where Δ*X* and *N* denotes cv residuals and the number of reference points, respectively.

### 2.3. Stability constants selection

Because of huge variations between experimental stability constants it was important that selected constants were measured under the same conditions (ionic strength, temperature), and preferably in the same laboratory. It was a bit surprising to find out that the most consistent constants were also the oldest, determined in 1950s. They were measured at *t* = 25 °C, and *I* = 0.01 or *I* → 0 mol L^−1^. If more than one experimental value was referred for a complex, we used mean value in further calculations. Moreover, lack of constants measured at these conditions forced us to include three constants, for Fe(Glycine), Fe(Glycine)_2_ and Fe(Valine), measured at *t* = 20 °C, *I* = 0.01 mol L^−1^. Unfortunately, appropriate log *K*_1_ andlog *β*_2_ values for Ni^2+^ complexes with valine were not found in the literature.

## 3. Results and Discussion

In this paper we tried to reproduce stability constants of chelates with bivalent metals constituting Irving-Williams order, whose stability grows monotonously (from Mn^2+^ to Cu^2+^), by means of valence connectivity index of the 3^rd^ order, ^3^*χ^v^*. Therefore, experimental values of stability constants of the metal complexes with four aliphatic *α*-amino acids (glycine, alanine, valine and leucine) were taken from the literature, Table 1. 

**Table 1:** Experimental stability constants for metal(II) chelates with *α*-amino acids.

Metal/Ligand	log *K*_1_	log *β*_2_	References
Cu/Glycine	8.57	15.63	[32,33,34,35]
Ni/Glycine	6.15	11.15	[32,33]
Co/Glycine	5.09	9.10	[32,33]
Fe/Glycine	4.30	7.80	[36]
Mn/Glycine	3.55	6.63	[32,33]
Cu/Alanine	8.41	15.21	[32,33,37,38]
Ni/Alanine	5.96	10.66	[33]
Co/Alanine	4.83	8.55	[32,33,39]
Fe/Alanine		7.30	[39]
Mn/Alanine	3.13	6.05	[32,33]
Cu/Valine	7.93	14.45	[32]
Co/Valine	4.57	8.24	[32]
Fe/Valine		6.80	[39]
Mn/Valine	2.84	5.56	[32]
Cu/Leucine	7.89	14.34	[32]
Ni/Leucine	5.62	10.18	[40]
Co/Leucine	4.52	8.16	[32,40]
Mn/Leucine	2.78	5.45	[32]

**Figure 2:**
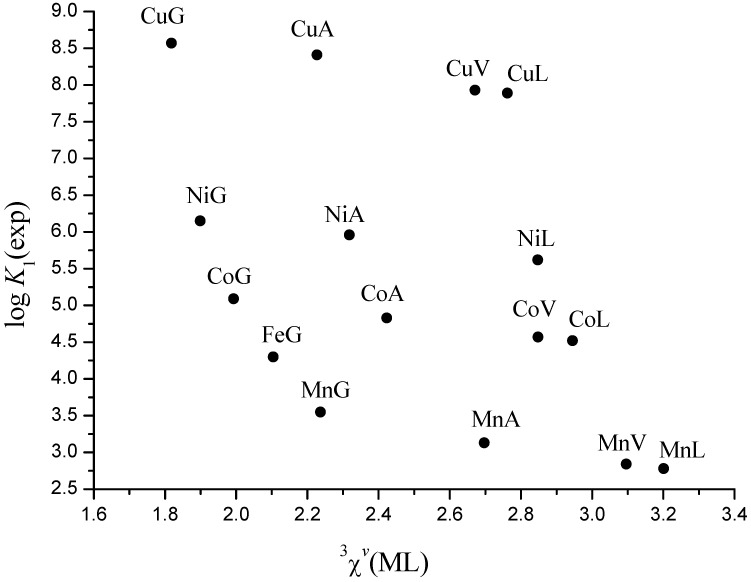
Experimental values of log *K*_1_* vs.* connectivity ^3^*χ^v^* index for Mn^2+^, Fe^2+^, Co^2+^, Ni^2+^, and Cu^2+^ complexes with glycine (G), alanine (A), valine (V) and leucine (L).

**Figure 3:**
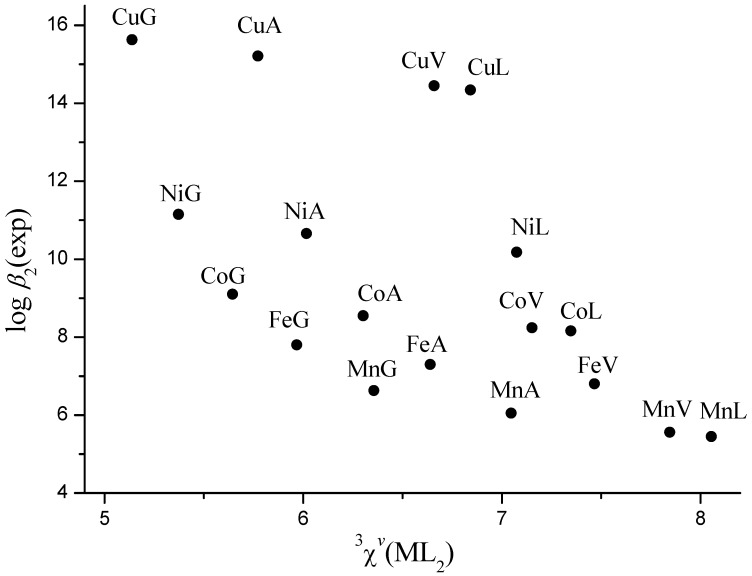
Experimental values of log *β*_2_* vs.* connectivity ^3^*χ^v^* index for Mn^2+^, Fe^2+^, Co^2+^, Ni^2+^, and Cu^2+^ complexes with glycine (G), alanine (A), valine (V) and leucine (L).

**Figure 4:**
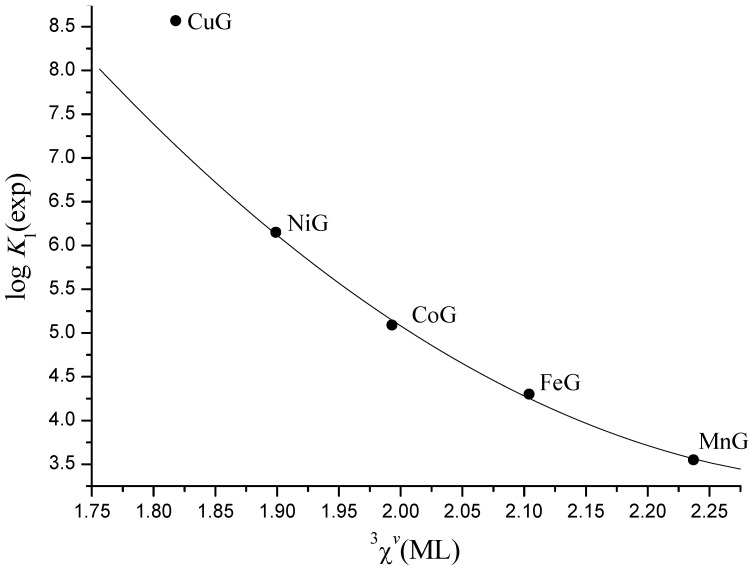
Quadratic dependence of log *K*_1_ of Mn^2+^, Fe^2+^, Co^2+^, and Ni^2+^ complexes with glycine (G) on connectivity index ^3^*χ^v^*.

It is evident (Figure 2 and Figure 3), that stability constants of the complexes with the same metal decrease linearly with ^3^*χ^v^* from glycine to leucine, as we have previously shown [17,30]. Figure 2 and Figure 3 also show that stability of *mono*- and *bis*-complexes decreases monotonously from Cu^2+^ to Mn^2+^ for complexes with the same ligand. However, the decrease is much more pronounced between Cu^2+^ and Ni^2+^ because Cu(II) is the strongest Lewis acid in Irwing-Williams order, and its complexes are therefore unusually stable [2]. Consequently, stability constants of Mn^2+^, Fe^2+^, Co^2+^, and Ni^2+^ complexes show quadratic dependence on ^3^*χ^v^*, but Cu(II) complexes considerably deviate from it (Figure 4). 

**Figure 5:**
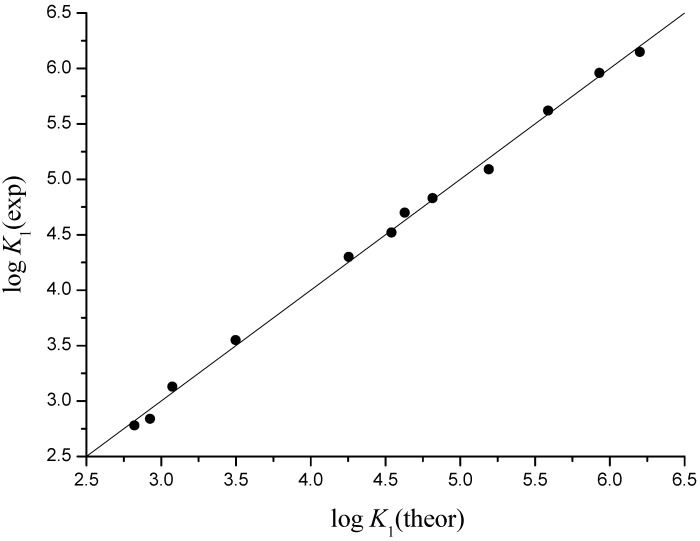
Experimental *vs.* theoretical (fit) log *K*_1_ for Mn^2+^, Fe^2+^, Co^2+^, and Ni^2+^ complexes with glycine, alanine, valine and leucine; *r* = 0.999, S.E._cv_ = 0.08.

**Figure 6:**
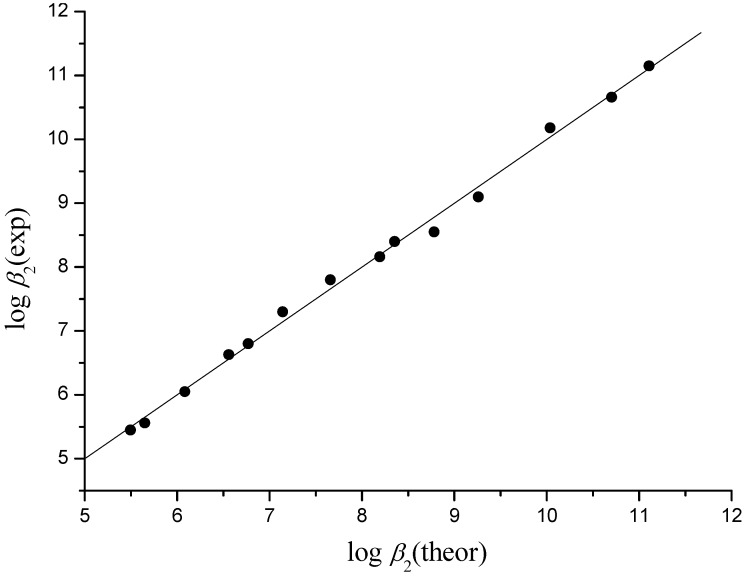
Experimental *vs.* theoretical (fit) log *β*_2_ for Mn^2+^, Fe^2+^, Co^2+^, and Ni^2+^ complexes with glycine, alanine, valine and leucine; *r* = 0.998, S.E._cv_ = 0.15.

Bearing this in mind we developed models for the simultaneous estimation of stability constants of Mn^2+^, Fe^2+^, Co^2+^ and Ni^2+^ complexes:

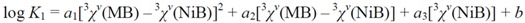
(6)

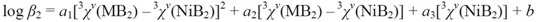
(7)
where ^3^*χ^v^*(NiB) and ^3^*χ^v^*(NiB_2_), stand for normalization along x axis in the first two terms, and for normalization along y axis in the third terms. Models gave standard error of cross validation S.E._cv_ = 0.08 and 0.15, and maximal cv error of 0.13 and 0.29 for log *K*_1_ and log *β*_2_, respectively (Table 1, Table 2 and Table 3, Figure 5 and Figure 6). 

**Table 2:** Regression models for the estimation of the stability constants *of mono*- and *bis*-complexes.

Eq.	*N*	Dependentvariable	Regression coefficients			Intercept (S.E.)	*r*	S.E.	S.E._cv_
			a_1_(S.E.)	a_2_(S.E.)	a_3_(S.E.)				
(6)	12	log *K*_1_	12.2(14)	−12.10(55)	−0.676(46)	7.49(12)	0.999	0.05	0.08
(7)	14	log *β*_ 2_	3.10(33)	−7.66(36)	−0.646(52)	14.58(33)	0.998	0.11	0.15

**Table 3:** Theoretical (cross validated) stability constants for metal(II) chelates with *α*-amino acids, and their ^3^*χ^v^* indices.

Metal/Ligand	log *K*_1_ (cv)	log *β*_2_ (cv)	^3^*χ^v^*(MB)	^3^*χ^v^*(MB_2_)
Ni/Glycine	6.21	11.11	1.90	5.37
Co/Glycine	5.18	9.25	1.99	5.65
Fe/Glycine	4.24	7.65	2.10	5.97
Mn/Glycine	3.51	6.58	2.24	6.36
Ni/Alanine	5.93	10.69	2.32	6.02
Co/Alanine	4.79	8.76	2.42	6.30
Fe/Alanine		7.13	2.55	6.64
Mn/Alanine	3.09	6.10	2.70	7.05
Ni/Valine			2.75	6.89
Co/Valine	4.59	8.32	2.85	7.15
Fe/Valine		6.74	2.96	7.47
Mn/Valine	2.91	5.64	3.10	7.85
Ni/Leucine	5.57	10.01	2.85	7.08
Co/Leucine	4.50	8.16	2.95	7.35
Mn/Leucine	2.81	5.48	3.20	8.06

## 4. Conclusions

Our regression models, Equations (6) and (7), clearly show that by using the connectivity index ^3^*χ^v^* it is possible to predict stability constants with an error 0.03–0.13 and 0.00–0.29 for log *K*_1_ and log *β*_2_, respectively, *i.e.* virtually within the limits of experimental error. Besides, maximal range of experimental values, 0.28 for log *K*_1_ of Co(Glycine) and 0.38 for log *β*_2_ of Co(Alanine)_2_, speaks strongly in favor of our model. All this leads to the conclusion that models for the prediction of stability constants based on connectivity index ^3^*χ^v^* could provide a chemist the simple and efficient tool for planning his experiments and discussing his results.
